# Suppressive chemoprophylaxis invites avoidable risk of serious illness caused by *Plasmodium vivax* malaria

**DOI:** 10.1016/j.tmaid.2013.01.002

**Published:** 2013-01

**Authors:** J. Kevin Baird

**Affiliations:** a Eijkman-Oxford Clinical Research Unit, Jalan Diponegoro No. 69, Jakarta 10430, Indonesia; b The Centre for Tropical Medicine, Nuffield Department of Medicine, University of Oxford, Oxford, United Kingdom

**Keywords:** Malaria, Causal prophylaxis, Primaquine, *Plasmodium vivax*

## Abstract

Despite inadequacy in preventing vivax malaria after travel, suppressive chemoprophylaxis has dominated travel medicine strategy since the advent of chloroquine in 1946. The lethal threat of falciparum malaria versus the perceived benign consequence of vivax malaria underpins this strategic posture. Recent evidence demonstrating vivax malaria as often pernicious should prompt reconsideration of that posture. Causal prophylaxis kills early developing forms of plasmodia in the liver, thus preventing attacks of falciparum and vivax malaria during travel and delayed onset vivax malaria following travel. Primaquine is the only available drug for this application, and has good evidence of safety, tolerability and efficacy in non-pregnant, G6PD-normal travelers. The primaquine label, however, carries no such indication. Risk of pernicious vivax malaria from all across the endemic regions of the globe, including much of sub-Saharan Africa, should raise consideration of daily primaquine during travel as the preferred front-line option for chemoprophylaxis against malaria in travelers.

## Acute pernicious vivax malaria

Malariologists, along with the workers in public health, science, and medicine who engage the malaria problem, have long accepted the notion that vivax malaria is almost always harmless. Consider the opening to the article, “*The Other Malaria*”, that appeared in the May 26th 2011 edition of *The Economist*, “*‘When is a disease not a disease?’ sounds like a childhood riddle. One answer, though might be, ‘when it is vivax malaria*.’” It goes on to explain, “Vivax *debilitates but it rarely kills*.” This view accurately reflects dominant expert opinion, but recent evidence from endemic zones shows approximately equal risk of severe or fatal illness with a diagnosis of *Plasmodium vivax* or *Plasmodium falciparum* among patients hospitalized with a primary diagnosis of malaria.^1^ Further, a comprehensive review of the historic evidence underpinning benign identity for *P. vivax* revealed nullifying weaknesses.^1^ Vivax malaria is certainly not inherently benign as long perceived, but it possesses the capacity to become pernicious. The broad perception of benign consequence may be the most dangerous aspect of vivax malaria – its threat to human life is not acknowledged, understood, or addressed.

**Fig. 1:**
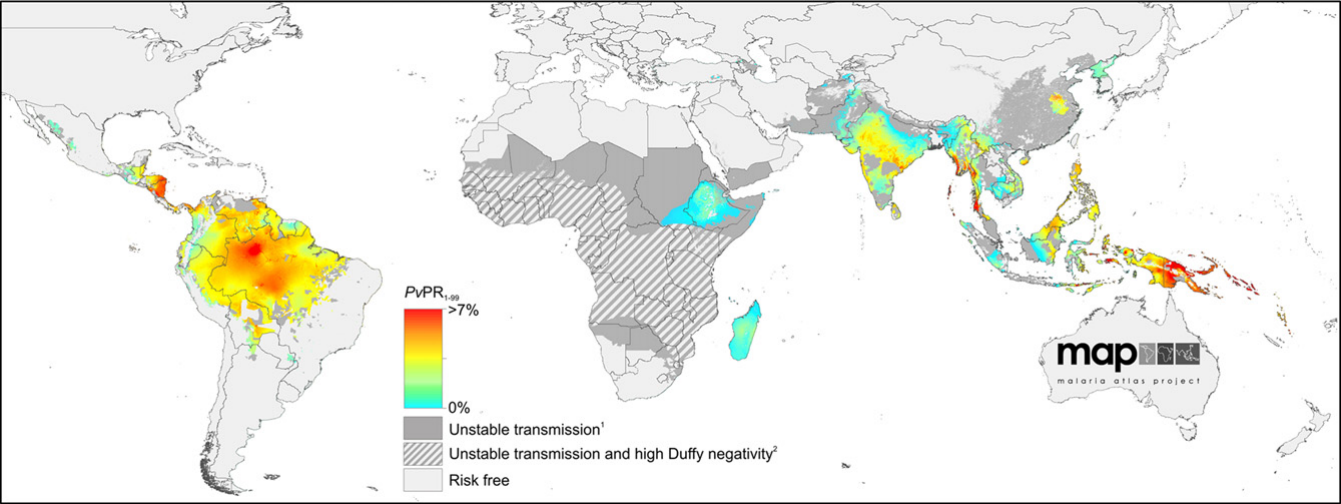
Global map of endemic vivax malaria, reprinted from Ref.^3^ with permission of the authors and *PLoS Neglected Tropical Diseases*.

**Fig. 2:**
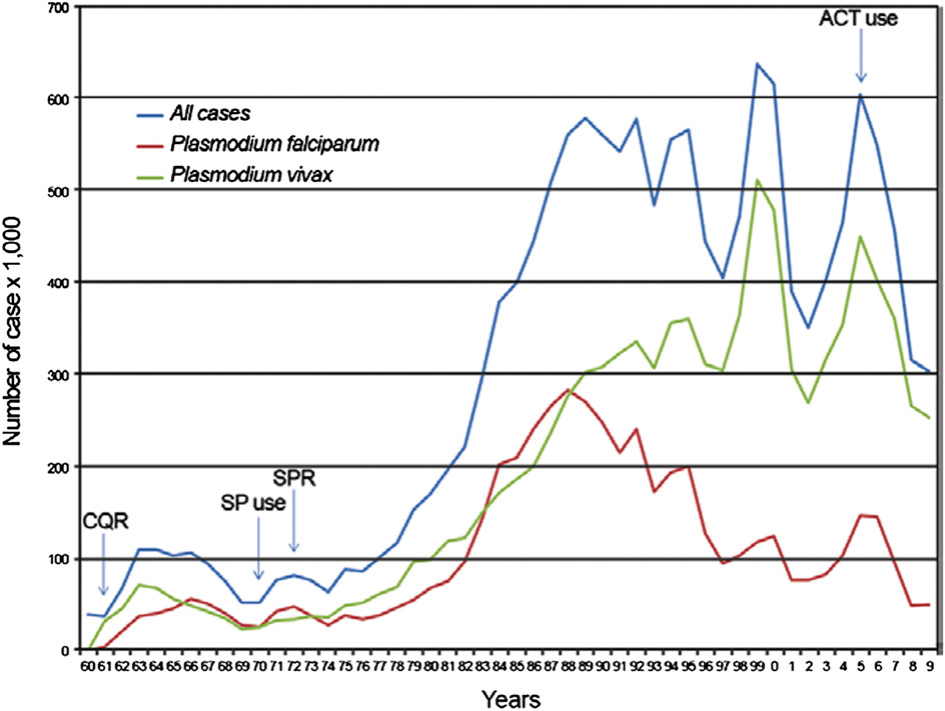
Burden of the malarias in Brazil since 1960, reprinted from Ref.^22^ with permission of the authors and *Mem Inst Oswaldo Cruz*. Endemic vivax malaria is less responsive to standard approaches to control and treatment.

**Fig. 3:**
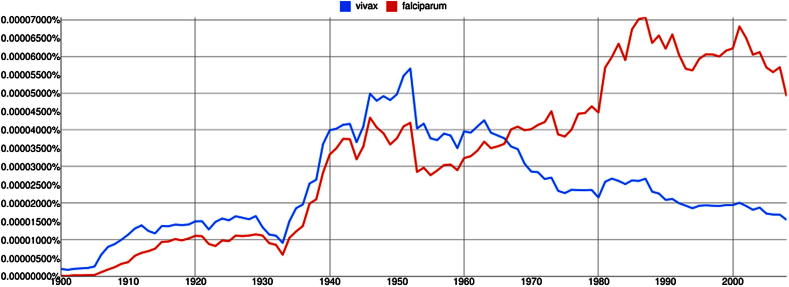
Top graph documents annual citations in published books as a percentage of total literature since 1900 (from http://books.google.come/ngrams courtesy of Lorenz von Seidlein, Darwin, Australia).

The estimated population at risk and the burden of disease with infection by *P. vivax* number several billion and a hundred million or more, respectively, and span the endemic world from the Korean peninsula to northern Argentina (Fig. 1).^2,3^ This species exhibits far less responsiveness to standard methods of control and prevention than *P. falciparum*, principally because the available chemotherapeutics are not fit for purpose against endemic vivax malaria (Fig. 2, from^4^). The benign identity assigned to *P. vivax* effectively arrested research on this infection sixty years ago (Fig. 3). Almost no research on therapies that prevent relapse has been done since the development of primaquine in American prisoner volunteers during and immediately after World War II.^5^ This neglect bears directly on questions of central importance to malaria as a problem of travel medicine.

## Traveler beware

As Fig. 1 illustrates, endemic vivax malaria occurs across much of the globe. Providers of travel medicine services obviously should carefully consider the threat to their patients posed by *P. vivax*. The decision on chemoprophylaxis versus personal protection is complex and based on often-inadequate data for rational assessments of real risk of exposure. This is also true for vivax malaria, but the false aegis of benign consequence versus deadly falciparum malaria may weigh inappropriately upon that decision-making process. In fact, vivax malaria not only carries risk of serious illness and death, but it also threatens in ways *P. falciparum* does not: 1) it is widely perceived as clinically inconsequential; 2) it may be much more prevalent than blood surveys like those underpinning Fig. 1 suggest; 3) the bulk of its threatening biomass in patients may occur beyond vascular sinuses and convenient assessment; 4) standard therapy for radical cure, chloroquine and primaquine, has been in continuous use since 1952 and suffers a number of serious pitfalls; 5) no alternative radical cure except quinine and primaquine has proven safety and efficacy; and 6) standard suppressive chemoprophylaxis does not prevent seeding of the liver with hypnozoites and risk of multiple relapses in the weeks and months following travel. In short, risk of infection by *P. vivax* is widespread, poses an inherent and insidious clinical threat, and it is notoriously difficult to prevent and treat.

## Travel medicine provider beware

A rational and practical strategy for avoiding acute vivax malaria would of course be useful to providers of travel medicine services and their patients. In considering chemoprophylactic strategy, the greater biological complexity of *P. vivax* compared to *P. falciparum* imposes important technical nuance. This species, along with *Plasmodium ovale*, places dormant forms in the liver that may provoke a clinical attack anywhere between 17 days and up to 3 years following patency of the primary infection.^6^ The risk of relapse varies from <10% to >90% depending upon where acquired, and the number of relapses ranges from 1 (e.g., Korean strains) to about 10 (e.g., tropical Asian strains).^7^ Despite the absence of prevalent *P. vivax* across much of Africa due to dominance of the Duffy negative phenotype (Fig. 1): much of eastern Africa has endemic vivax malaria and travelers to sub-Saharan Africa certainly acquire vivax malaria,^8^ and risk may occur on that continent even where the infection does not appear prevalent.^9^ Drugs used for suppressive chemoprophylaxis kill parasites after they have matured in the liver and emerged into the bloodstream (blood schizontocidal). These drugs will not prevent seeding of the liver with hypnozoites, nor impact the emergent blood stages months after travel. Inappropriate management of vivax malaria by underestimating its clinical threat, the risk of exposure to it, the enduring threat after travel, or by prescribing ineffective chemoprophylaxis may result in travelers suffering potentially dangerous clinical attacks that are difficult to manage and treat.

## Treatment provider beware

Difficult chemotherapeutic issues complicate management of patients with acute vivax malaria. Widespread resistance to chloroquine occurs^5^ and there is deep uncertainty regarding the efficacy and effectiveness of primaquine,^10^ especially when applied with modern blood schizontocides.^11^ The efficacy of primaquine against relapse requires an appropriate partner blood schizontocide, even when that partner alone has no impact upon hypnozoites, like chloroquine or quinine.^12^ In other words, the safety and efficacy of primaquine when partnered with any given blood schizontocide(s) may not be presumed -- it requires evidence that is very difficult to obtain and currently very scarce. Finally, recent studies suggest that a common mutant genotype of a CYP allele renders primaquine (combined with chloroquine) ineffective against relapse of *P. vivax*.

Clinical aspects of vivax malaria deepen the difficulty of its management. The benign identity inappropriately assigned to vivax malaria stemmed in large part from inherently low numbers of parasites in peripheral blood, typically at least an order of magnitude lower than those in patients infected by *P. falciparum*. Hypothetically, the low-grade parasitemias may represent only a small proportion of the threatening biomass: various physical, molecular, and behavioral characteristics of *P. vivax* suggest it is principally an infection of hemopoietic tissues rather than vascular sinuses.^1^ If that is proven to be true, a credible assessment of clinical threat may require examination of bone marrow aspirate in order to gauge parasite biomass and its clinical threat in patients. Examination of peripheral blood films alone may be dangerously misleading.

A recent trial of primaquine against relapse in Indonesian soldiers demonstrated a 0.5 mg/kg daily regimen for 14 days as 98% efficacious when combined with dihydroarte-misinin-piperaquine for radical cure.^13^ Primaquine therapy began on day 28 following patency for want of evidence demonstrating safety of co-administration. This trial nonetheless offers the first credible evidence for options to chloroquine-primaquine for radical cure since 1952. Work promising proof of safety with co-administration is in progress. Dihydroartemisinin-piperaquine combined with primaquine should be the preferred option where risk of resistance to chloroquine occurs, i.e., most endemic zones, especially Southeast Asia and Oceania.

## Poor chemoprophylaxis

The axiomatic superiority of prevention versus cure may be especially true for vivax malaria. Avoidance of the risks and difficulties in managing and treating patients with vivax malaria certainly emphasizes the importance of success in preventive measures. However, travel medicine doctrine embraces suppressive chemoprophylactic strategy that is unreliable against vivax malaria for the logical reasons already explained – and available evidence demonstrates this inadequacy. Schwartz and Regev-Yochay^14^ documented the relatively high risk of vivax malaria in the months following travel using suppressive rather than causal prophylaxis among Israeli travelers to eastern Africa: 6%, 53%, and 52% vivax malaria attack rates with primaquine, doxycycline, and mefloquine for prophylaxis, respectively, after more than 3 months following cessation of travel. Schwartz and colleagues^15^ described 35%–45% of travelers suffering malaria developing delayed onset (>2 months post-travel) malaria, and 90% or more of these were caused by *P. vivax* or *P. ovale*. More to the point, 62%–81% of these delayed onset cases had been using suppressive chemoprophylaxis as prescribed.

Suppressive prophylaxis remains the primary means of preventing malaria in travelers.^16^ The risk of relapse in travelers is supposedly managed by post-travel presumptive anti-relapse therapy (PART), i.e., a daily dose of 0.5 mg/kg primaquine for 14 days.^17^ However, as explained by Freedman,^18^ “*…presumptive antirelapse therapy is used infrequently in practice and only in patients with the most obvious and prolonged exposure to infective mosquitoes*”. One agency advises, “*…routine use of primaquine for prophylaxis* [post-travel PART] *is not recommended…*”.^19^ The US CDC advises^16^ on post-travel PART, *“Presumptive antirelapse therapy is generally indicated only for people who have had prolonged exposure in malaria endemic areas (such as missionaries or volunteers).”* The consequences of these practices include several hundred cases of delayed onset post-travel vivax malaria each year in the United States alone.^14,18^ In 2010 in the USA, 319 people were diagnosed with *P. vivax*, with 41% of those occurring more than a month after return from travel.^20^ Further, among the 946 patients known to have been hospitalized with a primary diagnosis of malaria in the USA, 95 had a diagnosis of *P. vivax* and 20% of those patients (*n* = 19) suffered severe illness^20^ – roughly the same rate of severe illness occurring in many hospitals in endemic zones.^1^ Does avoidance of the cost, inconvenience, and risk of post-travel PART merit these infections collateral to demonstrably poor chemoprophylaxis strategy? Few providers today, understanding vivax malaria as capable of a pernicious course, as it certainly is in travelers [see listing of case reports in reference #1], would consider permitting relapse in their patients an acceptable option.

Unfortunately, post-travel PART comes with more than cost, inconvenience and risk of toxicity pitfalls – there is no evidence of safety or efficacy when used following suppressive prophylaxis (with the certain exception of chloroquine and the possible exception of mefloquine). As explained elsewhere, the efficacy of primaquine against hypnozoites requires an appropriate partner drug in radical cure.^11,12^ The evidence for this, developed during the pivotal clinical trials of primaquine 65 years ago, became forgotten and mostly irrelevant in practice because the chloroquine partner for primaquine was the drug of choice in both chemoprophylaxis and treatment. Primaquine kills hypnozoites when partnered with chloroquine for either indication. However, the efficacy of primaquine when used after other suppressive chemoprophylactic drugs like doxycycline or atovaquone/proguanil has not been demonstrated. Indeed, good efficacy for primaquine may be doubtful when partnered with these relatively rapidly excreted drugs. When Alving et al.^21^ gave primaquine following rather than currently with daily quinine for therapy of acute vivax malaria, 15 of 19 versus 2 of 19 subjects, respectively, suffered relapse. Similar findings, reported before and after this report, all point to the same conclusion: primaquine requires an appropriate companion drug to achieve good efficacy at doses normally considered therapeutic.^11,12^

Available evidence strongly suggests that post-travel PART administered following travel using the most widely prescribed agents of malaria chemoprophylaxis may have no efficacy against relapse of *P. vivax*. This possibility taken with the general reluctance to even recommend the treatment, suggests that suppressive chemoprophylaxis may be reasonably expected to routinely fail against vivax malaria. The report from Schwartz and colleagues^15^ from nine years ago came with this conclusion, “*Agents that act on the liver phase of malaria parasites are needed for more effective prevention of malaria in travelers*.” The primary aim of this commentary in this especially relevant issue of *Travel Medicine & Infectious Diseases* is to revisit and emphasize this point against a backdrop of dangerous and difficult to manage and treat vivax malaria.

## Primaquine for primary prophylaxis

The good safety, tolerability and efficacy of daily primaquine for primary causal prophylaxis have been demonstrated in subjects considered good candidates for the drug (G6PD-normal, not pregnant).^14,22,23^ This may seem to contradict the already explained reliance of primaquine on appropriate partner drugs to prevent relapse, but it does not – daily primaquine during exposure to infection kills early developmental stages of the parasite in the liver rather than mature hypnozoites. The necessity of daily dosing of primaquine thus begins with travel and ends a few days following exposure to risk (evidence supports 5 days, but 2 or 3 days may suffice). The returned traveler may be assumed to be free of hypnozoites.

Although primaquine is certainly dangerous to pregnant women and patients with G6PD deficiency, it proved remarkably safe and well tolerated in other patients consuming 0.5 mg/kg daily for 16–50 weeks. Among non-pregnant and G6PD-normal subjects taking the recommended daily adult dose of 30 mg with a snack for as long as 50 weeks in a double-blinded and placebo-controlled trial, safety and tolerability was comparable to placebo.^24^ The well-known and rarely symptomatic met-hemoglobinemia of primaquine therapy (averaging about 6% met-hemoglobin after several days dosing) was not exacerbated with prolonged dosing and returned to normal levels within 2 weeks of cessation.^24,25^ Other studies reported similarly good safety and tolerability.^26,27^ One study in Colombian soldiers in the field reported discontinuation of primaquine prophylaxis in 3 of 122 subjects due to severe gastrointestinal distress; six others had mild to moderate distress.^28^

## Reluctance to use primaquine for primary prophylaxis

Apart from the caveat of contraindications for pregnant women and patients with G6PD deficiency, daily primaquine during travel seems the ideal chemoprophylactic solution for most of the endemic world. So why is primaquine not more widely recommended and used? Several factors must be considered in striving to answer this important question: 1) the long dominance of suppressive chemoprophylaxis in travel medicine doctrine; 2) the perception of vivax malaria as harmless; and 3) primaquine not having a registered therapeutic indication for primary prophylaxis.

As in many other areas of medicine and public health,^29,30^ the presumed harmlessness of vivax malaria relegated it to neglect in travel medicine. The virtually exclusive monopoly on chemoprophylaxis by suppressive drugs effective against falciparum malaria dates to the licensing of chloroquine in 1946. Risk of delayed onset vivax malaria after travel with suppressive prophylaxis seemed preferable to the inconvenience and complexity of post-travel PART with primaquine.^18,19^ Certainly the availability of good evidence of safety and efficacy of primaquine for primary prophylaxis for over a decade has yet to prompt real change in chemoprophylaxis strategy. Suppressive prophylactic drugs remain today, according to most authorities, first-, second-, and third-line options for chemoprophylaxis in travelers.^16–18^ Primaquine primary prophylaxis is either a last option, listed as a footnote, or not mentioned.^19^ One cannot know precisely what the makers of such recommendations weighed, but clinically inconsequential vivax malaria would appear to have been a key consideration to strategies favoring suppressive chemoprophylaxis with post-travel PART scarcely recommended or not at all.

The travel medicine community has been reluctant to prescribe primaquine for primary prophylaxis as a consequence of off-label use liability. Fifteen years ago efforts by the U.S. Department of Defense to have primary prophylaxis added to the primaquine label as an approved indication ended in frustration – no stakeholder was willing to bear the cost of doing so. Acknowledging the clinical threat of vivax malaria and the inadequacy of suppressive prophylaxis against it should spark renewed efforts to see the label for primaquine include an indication for primary prophylaxis. The travel medicine community, being the primary stakeholder in this issue, should engage in advocacy for regulatory action on this vitally important drug that brings sanctioned use as primary prophylaxis.

The view of primaquine casual prophylaxis as unsuitable for travel to Africa, aired in some travel medicine circles, may have created the false impression that it is ineffective against *P. falciparum*. The basis of that view would likely have been the presumably low risk of vivax malaria rather than a high risk of falciparum malaria. Primaquine exhibits relatively poor blood schizontocidal activity against *P. falciparum*, but this has no bearing on its activity in causal prophylaxis – killing early liver stages precludes the relevance of blood schizontocidal activity in chemoprophylaxis. Risk of “breakthrough” parasitemias with primaquine prophylaxis, as occur with suppressive prophylaxis, being more likely to advance more quickly to threatening illness is speculative and not supported by available data in patients.

Atovaquone-proguanil has causal prophylactic activity against *P. falciparum*, and this is the basis of the recommendation to consume it for 7 days rather than the usual 28 days for suppressive chemoprophylaxis.^30^ However, this activity against *P. vivax* or *P. ovale* has not been demonstrated^31^ and at least several cases suggest atovaquone-proguanil prophylaxis does not prevent formation of hypnozoites.^32–35^

## Due diligence

Today and for the foreseeable future, primaquine is the only widely available drug offering good evidence of safety, tolerability, and efficacy against primary infections of both *P. vivax* and *P. falciparum* during exposure, and against relapse of *P. vivax* in the months following exposure. This evidence, weighed against that showing unequivocal poor performance of suppressive prophylactic drugs against delayed onset vivax malaria in travelers – along with evidence of risk of vivax malaria across much of the globe – raises an obvious and important question for the travel medicine community: Is recommending and prescribing suppressive prophylaxis for any traveler the reckless option to primaquine causal prophylaxis?

## Disclosures

The author is an unpaid consultant to GlaxoSmithKline on the development of tafenoquine, a therapy intended to replace primaquine against relapse of vivax malaria. The author receives research support from the Medicines for Malaria Venture for clinical trials of primaquine therapy.

## Conflict of interest

None declared.

## References

[1] Baird J.K. (2013). Evidence and implications of mortality associated with acute *Plasmodium vivax* malaria. Clin Rev Microbiol.

[2] Gething P., Elyazar I.R.F., Moyes C.L., Smith D.L., Battle K.E., Guerra C.A. (2012). A long neglected world malaria map: *Plasmodium vivax* endemicity in 2010. PLoS Negl Trop Dis.

[3] Guerra C.A., Howes R.E., Patil A.P., Gething P.W., Van Boeckel T.P., Temperley W.H. (2010). The international limits and population at risk of *Plasmodium vivax* transmission. PLoS Negl Trop Dis.

[4] Gamma B.E., Lacerda M.V., Daniel-Ribero C.T., Ferreira-da-Cruz F. (2011). Chemoresistance of *Plasmodium falciparum* and *Plasmodium vivax* parasites in Brazil: consequences on disease morbidity and control. Mem Inst Oswaldo Cruz.

[5] Baird J.K. (2009). Resistance to therapies for infection by *Plasmodium vivax*. Clin Microbiol Rev.

[6] White N.J. (2011). Determinants of relapse periodicity in *Plasmodium vivax* malaria. Malar J.

[7] Kitchen S.F., Boyd M.F. (1949). Vivax malaria. Malariology.

[8] Leder K., Black J., O’Brien D., Greenwood Z., Kain K.C., Schwartz E. (2004). Malaria in travelers: a review of the GeoSentinel surveillance network. Clin Infect Dis.

[9] Culleton R., Ndounga M., Zeyrek F.Y., Coban C., Casimiro P.N., Takeo S. (2009). Evidence for the transmission of *Plasmodium vivax* in the Republic of Congo, West Central Africa. J Infect Dis.

[10] John G.K., Douglas N.M., von Seidlein L., Nosten F., Baird J.K., White N.J. (2012). Primaquine radical cure of *Plasmodium vivax*: a critical review of the literature. Malar J.

[11] Baird J.K. (2011). Resistance to chloroquine unhinges vivax malaria therapeutics. Antimicrob Agents Chemother.

[12] Baird J.K., Maguire J.D., Price R.N., Hay S.I., Rollinson D. (2012). Diagnosis and treatment of Plasmodium vivax malaria. The epidemiology of *Plasmodium vivax*: history, hiatus, and hubris.

[13] Sutanto I., Tjahjono B., Basri H., Taylor W.R., Putri F.A., Meilia R.A. (2013). Randomized, open label trial of primaquine against vivax malaria relapse in Indonesia. Antimicrob Agents Chemother.

[14] Schwartz E., Regev-Yochay G. (1999). Primaquine as prophylaxis for malaria for nonimmune travelers: a comparision with mefloquine and doxycycline. Clin Infect Dis.

[15] Schwartz E., Parise M., Kozarsky P., Cetron M. (2003). Delayed onset of malaria – implications for chemoprophylaxis in travelers. N Engl J Med.

[16] Arguin P.M., Mali S. (2012). Malaria. Infectious diseases related to travel. The yellow book. US centers for disease control.

[17] Treatment Guidelines (2010). Drugs for parasitic infections. Med Lett.

[18] Freedman D.O. (2008). Malaria prevention in short-term travelers. New Engl J Med.

[19] Chiodini P., Hill D., Lalloo D., Lea G., Walker E., Whitty C. (January 2007). Guidelines for malaria prevention in travelers from the United Kingdom.

[20] Mali S., Kachur S.P., Arguin P.M. (2010). Malaria surveillance—United States. MMWR Surveill Summ.

[21] Alving A.S., Arnold J., Hockwald R.S., Clayman C.B., Dern R.J., Beutler E. (1955). Potentiation of the curative action of primaquine in vivax malaria by quinine and chloroquine. J Lab Clin Med.

[22] Baird J.K., Fryauff D.J., Hoffman S.L. (2003). Primaquine for prevention of malaria in travelers. Clin Infect Dis.

[23] Hill D.R., Baird J.K., Parise M.E., Lewis L.S., Ryan E.T., Magill A.J. (2006). Primaquine: report from CDC expert meeting on malaria chemoprophylaxis I. Am J Trop Med Hyg.

[24] Fryauff D.J., Baird J.K., Basri H., Sumawinata I., Purnomo, Richie T.L. (1995). Randomized placebo-controlled trial of primaquine for prophylaxis of falciparum and vivax malaria. Lancet.

[25] Baird J.K., Lacy M.D., Basri H., Barcus M.J., Maguire J.D., Bangs M.J. (2001). Randomized, parallel placebo-controlled trial of primaquine for malaria prophylaxis in Papua, Indonesia. Clin Infect Dis.

[26] Baird J.K., Fryauff D.J., Basri H., Bangs M.J., Subianto B., Wiady I. (1995). Primaquine for prophylaxis against malaria among nonimmune transmigrants in Irian Jaya, Indonesia. Am J Trop Med Hyg.

[27] Weiss W.R., Oloo A.J., Johnson A., Koech D., Hoffman S.L. (1995). Daily primaquine effective for prophylaxis against falciparum malaria in Kenya: comparison with mefloquine, doxycyline, and chloroquine plus proguanil. J Infect Dis.

[28] Soto J., Toledo J., Rodriguez M., Sanchez J., Herrera R., Padilla J. (1998). Primaquine prophylaxis against malaria in nonimmune Colombian soldiers: efficacy and toxicity: a randomized, double-blind, placebo-controlled trial. Ann Intern Med.

[29] Baird J.K. (2007). Neglect of *Plasmodium vivax* malaria. Trends Parasitol.

[30] Price R.N., Tjitra E., Guerra C.A., Yeung S., White N.J., Anstey N.M. (2007). Vivax malaria: neglected and not benign. Am J Trop Med Hyg.

[31] Berman J.D., Nielson R., Chulay J.D., Dowler M., Kain K.C., Kester K.E. (2001). Causal prophylactic efficacy of atovaquone-proguanil (malarone) in human challenge model. Trans Roy Soc Trop Med Hyg.

[32] Maguire J.D., Llewellyn D.M. (2007). Relapsing vivax malaria after 6 months of daily atovaquone/proguanil in Afghanistan: the case for expanded use of primaquine as a causal prophylactic. J Travel Med.

[33] Povinelli L., Monson T.A., Fox B.C., Parise M.E., Morrisey J.M., Vaidya A.B. (2003). *Plasmodium vivax* malaria in spite of atovaquone/proguanil (Malarone) prophylaxis. J Travel Med.

[34] Gallien S., Taieb F., Schlemmer F., Lagrange-Xelot M., Atlan A., Sarfati C. (2008). Failure of atovaquone/proguanil to prevent *Plasmodium ovale* malaria in traveler returning from Cameroon. Travel Med Infect Dis.

[35] Jimenez B.C., Navarro M., Huerga H., Lopez-Roman E., Mendoza A., Lopez-Velez R. (2008). Tertian malaria (*Plasmodium vivax* and *Plasmodium ovale*) in two travelers despite atovaquone-proguanil prophylaxis. J Travel Med.

